# Mixed Effects of Deep Brain Stimulation on Depressive Symptomatology in Parkinson’s Disease: A Review of Randomized Clinical Trials

**DOI:** 10.3389/fneur.2014.00154

**Published:** 2014-08-11

**Authors:** N. Simay Gökbayrak, Irene Piryatinsky, Rebecca A. Gavett, Omar J. Ahmed

**Affiliations:** ^1^Department of Psychology, University of Rhode Island, Kingston, RI, USA; ^2^Memory and Aging Program, Butler Hospital, The Warren Alpert Medical School of Brown University, Providence, RI, USA; ^3^Department of Neurology, Massachusetts General Hospital, Harvard Medical School, Boston, MA, USA

**Keywords:** Parkinson’s disease, deep brain stimulation, depression, DBS, randomized clinical trial

## Abstract

Although ~50% of patients with Parkinson’s disease (PD) experience depression, treatment for this important and debilitating comorbidity is relatively understudied. Deep brain stimulation (DBS) has been increasingly utilized for the management of tremors in progressive PD. Several preliminary studies have shown the potential benefit of DBS for non-motor PD symptoms such as depression. Here, we critically evaluate seven recent randomized clinical trials of the effectiveness of DBS in reducing depressive symptomatology among individuals with PD. Findings are mixed for the effectiveness of DBS as a treatment for depression in PD. Our review suggests that this is due, in large part, to the anatomical and methodological variation across the DBS studies. We provide a comprehensive discussion of these variations and highlight the need to conduct larger, more controlled studies aimed specifically at evaluating the treatment of depression in PD patients.

## Introduction

Parkinson’s disease (PD) is a neurodegenerative illness found in 1–2% of individuals over age 65 in the United States (1). It is estimated that 30–70% of PD patients experience comorbid depression (2, 3). Symptoms of depression can begin at the initial onset of motor symptoms in PD (i.e., resting tremor, akinesia, bradykinesia, muscular rigidity, shuffling steps, and postural instability) and progress over time with substantial negative effects on overall well-being (4, 5). Depression has been linked to falls, disease progression, and negative views of PD (4). Until recently, there has been little awareness in the medical community regarding the severity and prevalence of depression in PD, and as a result, depression in this population remains under-treated. Furthermore, depression and PD have overlapping symptoms that render them difficult to identify and treat (6). For instance, symptoms such as “facial masking” in PD, which limits expression of emotions, may appear to be like flat affect, a characteristic of depression. Bradykinesia due to PD could also be viewed as a feature of depression (6).

The etiology of depression in PD is unclear. One school of thought is that depression is a result of the progressive PD experience. However, Eskow Jaunarajs et al. (7) have suggested that higher comorbidity between depression and PD compared to other neurodegenerative illnesses such as multiple sclerosis (8) and Alzheimer’s disease (9) indicates that there are other underlying physiological factors in play. Even when motor symptoms improve with treatment, patients frequently continue to endorse symptoms of depression (10). Depression is frequently the presenting symptom before significant motor symptoms are observed (11, 12). As such, it is difficult to determine whether depression is a consequence of the process of PD or of the emotional repercussions of the disease.

Large randomized clinical trials (RCTs) examining treatment effects for depression in PD patients are scarce (13). Selective serotonin reuptake inhibitors (SSRIs) and tricyclic antidepressants, the most commonly used medications for depression in PD (13), may have beneficial effects. However, SSRIs may also increase motor symptoms and tricyclics may contribute to other non-motor symptoms such as delirium and memory difficulties (13). Another form of treatment – electroconvulsive therapy (ECT) – has been used to treat psychosis, refractory depression, and PD-associated movement disorders (14); however, ECT has been found to cause episodic confusion and worsen dyskinesia (15). Based on the effectiveness of talk therapy for depression, such an approach could be beneficial to manage depression in PD. However, systematic psychotherapeutic treatment options tailored for depression in PD appear to have not been rigorously researched (6).

The treatment of depression in PD precludes a systematic actuarial approach and rather has been found to be based on individual clinical opinion (13). The primary motor symptoms have been treated with levodopa (l-DOPA) and more invasive treatments such as deep brain stimulation (DBS). l-DOPA, a dopamine agonist has been widely accepted as the leading medical treatment for the motor symptoms of PD; nevertheless, it is often limited in controlling progressive symptoms related to “gait, balance, speech, swallowing, and cognition” in refractory PD (16).

As a result, DBS has been increasingly tested and utilized for the management of tremors in progressive PD (17–19). Several recent preliminary studies have shown the potential for short-term utility (i.e., effects lasting 6–12 months post-surgery) of DBS in PD for depression (20–24). Despite these initial findings, all of the studies were limited for several reasons including small sample sizes, a case or cohort design, or a lack of randomization and further longitudinal follow-up. Given that RCTs are regarded as the benchmark for informing subsequent clinical treatment, the purpose of this review is to evaluate the most recent RCTs of the effectiveness of DBS in reducing depressive symptomatology among individuals with PD.

## Literature Review

### Physiological bases of Parkinson’s disease

Parkinson’s disease is primarily associated with the loss of dopaminergic cells in the substantia nigra pars compacta (SNPC), located in the basal ganglia. The SNPC dopaminergic projections regulate both the direct and indirect motor-control pathways in the basal ganglia (25). When these SNPC dopaminergic neurons are lost, the output of the basal ganglia increases, leading to altered activity in the thalamus and neocortex (25), and resulting in the motor deficits typically observed in PD (26).

Levodopa increases the diminished dopamine concentrations in the basal ganglia (27), which explains why it is the most widely used PD medication. However, it can have a multitude of side effects and poses a risk for developing additional motor-related disabilities (28). Furthermore, as the disease progresses, l-DOPA treatment tends to lose its efficacy, while a number of complications (such as dyskinesia) may develop, probably due to progressive dopaminergic neuronal loss (28). Such complications occur in approximately half of the patients 5 years after treatment initiation and in ~80% of patients after 10 years of treatment (29, 30). Dopamine loss may also be involved in the onset of depression in PD (7). Clinical findings indicate that l-DOPA treatment does not necessarily improve mood and, in fact, may exacerbate mood symptoms (7) by further disturbing the function of the norepinephrine and serotonin systems, already affected by PD pathology. Eskow Jaunarajs and colleagues (7) reviewed four studies (31–34) with samples ranging from 23 to 422 patients in the latter realm. Findings indicated that patients undergoing l-DOPA treatment remained depressed or did not see a significant improvement in their depression. Several other studies have also indicated that PD patients with depression are more likely to receive higher doses of l-DOPA compared to PD patients without depression (35, 36). In summary, although l-DOPA is essential for managing motor symptoms, increases in depression may be a potential consequence of this treatment in patients with PD.

### Deep brain stimulation

Deep brain stimulation is a surgical treatment in which one or more electrodes are implanted in the basal ganglia, typically targeting either the subthalamic nucleus (STN) (37) or the globus pallidus interna (GPi) (38). The electrodes are connected to a stimulator, which delivers high-frequency electrical pulses in order to alter patterns of neuronal signaling within the targeted region (16).

Imaging and clinical studies on STN DBS show changes in neuronal activity in the frontal lobe and the limbic system (39) and changes in frontal lobe function, respectively (40, 41). As such, in addition to improving motor function, STN DBS can affect frontal-lobe-dependent executive functioning, attention, emotions, and memory, all functions negatively impacted by a depressive syndrome (23).

### Clinical findings

Overall, the preliminary findings regarding the effectiveness of DBS surgery on mood in PD have been mixed. A meta-analysis (42) reviewing 82 studies evaluating behavioral outcomes in PD patients after undergoing STN DBS surgery found that 8% of the total sample (*N* = 1398) had depression after DBS treatment. Depression typically reduced after “adequate” psychotropic treatment (the specifics of treatment were not provided by the authors); however, 4% of the depressed patients subsequently attempted suicide (42). Therefore, a patient may have still met criteria for depression post-treatment but also have experienced a substantial decrease in symptoms. Although these small differences in symptom reduction may not reach statistical significance, clinically speaking, these differences may still have a positive impact on the patient’s functioning. The authors did not report outcome measures or potential premorbid depression levels so it is important to use caution in interpreting these findings.

In a structured review of 23 cohort and case studies found that depression reduced or remained unchanged after STN DBS within the first year of surgery, whereas little effect on depression level was observed after any of the other procedures [i.e., Ref. (23)]. A separate study assessed 33 patients, after undergoing STN DBS, finding that symptoms of depression decreased significantly (*p* = 0.007) post-surgery up until the first year and then returning to pre-surgery level at year 3 (43). In contrast, Castelli and colleagues (44) found that depression at the 3-year follow-up visit did not improve in the STN DBS group compared to an l-DOPA control group who did not undergo DBS surgery. Collectively, non-RCT findings potentially indicate short-term mood improvement after DBS.

## Methods

A literature review search was conducted via Google Scholar and PubMed through March 2013 employing the following combination of key words: DBS, depression, and Parkinson’s; DBS, mood, and Parkinson’s; DBS, Parkinson’s, and psychiatric outcomes; clinical trials, DBS, depression, and Parkinson’s. Inclusion criteria comprise studies written in English and studies looking at symptoms of depression as one of their outcome measures. Exclusion criteria included any studies that were non-RCTs. Seven original research articles met the inclusion criteria and were considered for the review (45–51).

## Results

Four research groups (based in Argentina, Germany/Austria, and the United States) conducted the seven studies (*N* = 889) reviewed. Table 1 reports study characteristics and findings.

**Table 1:** **Randomized clinical trials included in the present review**.

Author	Location	Sample size	Intervention	Outcome measure	Findings	*p* Values and effect size
Okun et al. (45)	USA	110	Unilateral STN DBS vs. unilateral GPi DBS Assessment at baseline and 6-months post-surgery	BDI	Those with pre-DBS depression had significantly higher BDI scores than non-depressive group after both treatments	*p* = 0.04
Follett et al. (46)	USA	299	Bilateral STN DBS vs. bilateral GPi DBS Assessment at baseline and 24-months post-surgery	BDI	Depression improved for GPi and worsened for STN	*p* = 0.02
Okun et al. (47)	USA	45	Unilateral STN DBS vs. unilateral GPi DBS Assessment at baseline and 6-months post-surgery	BDI	No differences between STN and GPi Overall improvement in both groups	*p* = 0.30 ns
Weaver et al. (48)	USA	255	Medical treatment vs. Bilateral STN DBS Assessment at baseline and 6-months post-tx	BDI	No significant group differences Both groups indicated minimal symptom reduction	*p* = 0.22
Zahodne et al. (49)	USA	42	Unilateral STN DBS vs. unilateral GPi DBS Assessment at baseline and 6-months follow-up	BDI	No significant group differences	ns
Merello et al. (50)	ARG	15	Bilateral STN DBS vs. bilateral subthalamotomy (BL) vs. unilateral subthalamotomy plus correlateral implementation STN DBS Assessment at 1-month before, and 6 and 12-months post-surgery	HAM-D	No significant group differences	*p* < 0.7, 0.9, 0.8 (pre-surgery, 6, and 12 months post-surgery, respectively)
Witt et al. (51)	GER	123	Medical treatment vs. bilateral STN DBS Assessment at baseline and 6-months post-tx	BDI MDRS	Depression improved “slightly” for DBS group on both measures	*p* = 0.06, Cohen’s *d* = 0.2 *p* = 0.07, Cohen’s *d* = 0.3

*BDI, Beck depression inventory; HAM-D, Hamilton depression scale; MDRS, Montgomery–Asberg depression rating scale; ARG, Argentina; GER, Germany; USA, United States; STN DBS, subthalamic nucleus deep brain stimulation; GPi DBS, globus pallidus pars interna deep brain stimulation; ns, not significant*.

In the Weaver et al. study (48), those who were randomized to the DBS group were then again randomized to receive either STN (*n* = 60) or GPi (*n* = 61). The study found statistically non-significant reductions of depression scores indicated by a 1.5 mean reduction in the medical treatment group (*n* = 134) compared to a 0.4 point mean reduction in the totality of the DBS group. Separate scores for the STN and GPi DBS groups were not provided. At the 6-month follow-up, both DBS groups reported more depression compared to best medical treatment group.

In contrast, Witt and colleagues (51) found that depression, although not statistically significant, minimally improved in the bilateral STN DBS group (*n* = 63) relative to the medical treatment group (*n* = 60) indicated by small effects on both self-reported and clinician-administered measures of depression at 6-months follow-up. Moreover, 13% of the DBS group and 10% of the medical treatment group experienced severe psychiatric adverse events that included suicide, “death during a psychotic episode” (the specifics were not provided), psychosis, and depression. Of those, four DBS participants endorsed depression but their episode was in remission by follow-up.

In a single blind study with the largest sample (46) compared to the other reviewed studies [and with the same recruitment methods described in Ref. (48)], those in the bilateral GPi DBS group (*N* = 152) had a mean reduction of 0.6 points whereas those in the STN DBS group (*N* = 147) had a mean increase of 1.3 points on symptoms of depression at the 24-month follow-up. One patient who underwent GPi DBS committed suicide, two patients undergoing STN DBS attempted suicide, and one other patient undergoing STN DBS had suicidal ideation. There were no significant group differences in terms of experiencing such adverse events.

In another study (47), both unilateral STN DBS (*N* = 22) and GPi DBS (*N* = 23) groups showed an overall mean reduction in symptoms of depression (M = −3.7, SD = 5.9); however, there were no significant differences between treatment groups at the 7-month follow-up. Mood-related adverse events including anxiety, irritability, aggressiveness, obsessive–compulsive symptoms, manic symptoms, and decreased motivation were more frequent in the STN group (*N* = 75) vs. the GPi group (*N* = 45), indicating that, overall, patients were experiencing multiple events.

In a retrospective study, Okun et al. (45) compared those with a diagnosis or history of depression (*N* = 40) and those without premorbid depression (*N* = 70) at a 6-month follow-up who had either received unilateral STN DBS or unilateral GPi DBS. Patients with premorbid depression were significantly more depressed at follow-up compared to those with no prior depression. Differences in mood symptoms at follow-up were not reported between STN DBS and GPi DBS groups.

The fourth and last unilateral STN DBS (*N* = 20) vs. unilateral GPi DBS (*N* = 22) study (49) found that for both treatment groups, depression significantly improved at the 6-month follow-up visit (*p* < 0.001). The GPi group showed a slightly larger mean reduction (−4.6 points) compared to that showed by the STN group (−2.6 points), but there were no significant group differences. Participants were recruited as part of a larger trial (47) described earlier.

In the final study of the present review (50), the three treatment groups included bilateral STN DBS (*N* = 5), bilateral subthalamotomy (BL) (*N* = 5), and unilateral subthalamotomy plus contralateral STN DBS (L/S) (*N* = 5). There were no treatment effects on symptoms of depression at any follow-up time point. Reportedly, two patients in the bilateral STN group presented with irritability, excitation, paranoia, and insomnia post-surgery but returned to baseline level after stimulation adjustments were made. In addition, one other patient presented with “severe apathy” that necessitated ongoing treatment.

## Discussion

Overall, findings are mixed with regards to the effectiveness of DBS as a treatment for depression in PD. Furthermore, it is unclear whether treatment effects are maintained long-term given the paucity of longitudinal research exceeding 1-year of follow-up.

Limited findings based on the present review and previous studies (52–54) indicated that GPi DBS may appear to be slightly safer compared to STN DBS because mood-related adverse events including suicide appear to be less common. An earlier pilot study (24) evaluated nine patients (five underwent unilateral STN DBS and four underwent unilateral GPi DBS). Stimulation at the site of optimal motor performance was linked to mood symptom reduction in both DBS treatments (24). However, overall, the GPi DBS group showed slightly increased symptoms compared to the STN DBS group (24). In contrast, Follett and colleagues (46) reported that GPi DBS minimally improved depression symptoms whereas STN minimally worsened it. One hypothesis for this difference in mood outcome relates to their anatomical size. The STN is smaller than the GPi, and, as a result, may be more vulnerable to surgical injury leading to a greater likelihood of post-surgery mood-related issues (47). However, differences among the fibers of passage coursing near the STN vs. GPi may also help to explain the differences between stimulation of the two regions.

Certainly, it is concerning that studies evaluating adverse events occurring in STN DBS have reported suicide attempts after surgery. One international multi-center review (55) found that retrospectively observed suicide rates after DBS STN surgery were higher than the expected age, gender, and country-adjusted suicide rates and continued to be high at the fourth post-surgery year (55). The most significant predictor of attempted and completed suicide was found to be post-surgery depression (55). As such, those individuals were more likely to have had pre-surgery depression as well (55). Mood-related complications after DBS are unclear and multiple confounding factors are potentially involved, including pre-DBS psychiatric symptoms (56), the surgery itself (57), the different types of electrical stimulation (58), adjustments in psychosocial functioning post-DBS (59), and the disease progression of PD (60).

### Strengths

There were several consistencies in the reviewed studies. Specifically, pre and post-treatment change in symptoms of depression were reported. All studies were blinded, RCTs with at least one follow-up time point and all studies with the exception of one included the Beck Depression Inventory as their outcome measure. The reviewed studies show preliminary evidence toward fine-tuning DBS surgery to help improve depressive symptoms that are highly associated with a debilitating disease, thus, providing hope for improving quality of life.

### Limitations

The results from the existing RCTs should be interpreted with caution because of primarily methodological limitations including small sample sizes, relatively short follow-up (6–12 months), and possible publication bias due to scant research in this area. Depression can have various etiologies. Given that all reviewed studies were part of larger clinical trials, and assessing mood symptoms typically was a secondary goal of the studies, previous diagnosis of depression and psychotropic medication history were not diligently reported. Also, none of the studies examined the use of psychotropic medication, talk therapy, or ECT as control or comparison groups, treatments that have been found to be effective with stand-alone depression.

Other possible reasons for the variation in the results may be due to the different levels of l-DOPA used at different time points between studies that may have impacted depression as well as the inconsistency of the brain regions stimulated by DBS. By nature of the surgery, surrounding brain regions such as the lateral hypothalamus and ventral tegmentum connected to the limbic system can be affected by the electrical stimulation and, hence, affect those areas that play a role in processing emotion (61). Additionally, the axons of neurons in distant cortical regions that send projections to the STN can also be activated by the high-frequency electrical pulses used in DBS (62). This highlights the importance of systematically dissecting the precise neuronal targets of DBS.

### Future research

Given the mixed findings and limitations in the research conducted so far, it is problematic to conclude what the current best practice should entail with regards to DBS surgery. There is emerging evidence suggest that the DBS target choice may be tailored to individual patient needs (16). When depression is of concern, the benefits associated with GPi stimulation may potentially outweigh the risks associated with STN DBS. As more RCTs are conducted, it will enable tailored target selection based on need and risk profiles (16).

Irrespective of etiology, depression should be consistently evaluated and treated by health care providers. One approach is to measure depression prior to treatment with PD medications and then measure the change in depression after PD medications have been administered (6). Assessing reliabilities in clinical response to depression in PD could allow some indication of when to treat depression as part of PD itself and when to treat depression as a separate syndrome (6).

Furthermore, one important clinical recommendation is, in addition to neuropsychological evaluation, an initial structured psychiatric assessment should be conducted for each newly diagnosed PD patient to serve as a baseline that can later be utilized if the patient does indeed opt for DBS.

Future interventions should aim to enroll larger sample sizes, control for premorbid/pre-surgery mood disorders, and consistently compare both within group variability and between group variability. Interventions should provide longitudinal follow-up visits after surgery to examine long-term protective effects of DBS.

A final thought is that a shift in perspective of depression in PD in the medical field is warranted. Thus, a comprehensive assessment of symptoms of depression guided by current research should be conducted when working with individuals with PD to better inform treatment protocols.

## Conflict of Interest Statement

The authors declare that the research was conducted in the absence of any commercial or financial relationships that could be construed as a potential conflict of interest.
